# The Impact of Black Race on Pneumonia Severity Scoring Systems

**DOI:** 10.1016/j.chpulm.2026.100234

**Published:** 2026-02-05

**Authors:** James Bradley, Matthew Wallace, Thomas R. Chandler, Stephen Furmanek, Julio Ramirez, Rodrigo Cavallazzi

**Affiliations:** a Division of Pulmonary, Critical Care Medicine, and Sleep Disorders, University of Louisville School of Medicine, Louisville, KY; b Norton Infectious Diseases Institute, Norton Healthcare, Louisville, KY

**Keywords:** community-acquired pneumonia, CURB-65, pneumonia, Pneumonia Severity Index, race, severe community-acquired pneumonia

## Abstract

**Background:**

Racial disparities disproportionately affect the morbidity and mortality of Black patients with community-acquired pneumonia (CAP). Some scoring systems used in the ICU (eg, Sequential Organ Failure Assessment, APACHE IVa) overpredict mortality in Black patients. However, to our knowledge, the performance of pneumonia severity scoring systems in White and Black adult patients has never been comprehensively evaluated.

**Research Question:**

Does Black race differentially impact the ability of pneumonia severity scoring systems to predict in-hospital mortality, direct ICU admission, or identify severe CAP?

**Study Design and Methods:**

This was a secondary analysis of 2 prospective cohorts of patients with SARS-CoV-2 CAP and non-SARS-CoV-2 CAP from 8 adult hospitals in Louisville, Kentucky. The following pneumonia severity scores were assessed: Pneumonia Severity Index; CURB-65; Confusion, Oxygenation, Respiratory Rate, and Blood Pressure scale; American Thoracic Society/Infectious Diseases Society of America (ATS/IDSA) 2007 criteria; SMART-COP; Severe Community-Acquired Pneumonia (SCAP) score; and Risk of Early Admission to ICU Index.

**Results:**

For Black and White patients with CAP, the in-hospital mortality rate was 5.6% vs 7.5% (*P* = .007), and the direct ICU admission rate was 15.2% vs 17.0% (*P* = .082). All pneumonia severity scores predicted in-hospital mortality equally in White and Black patients except the ATS/IDSA 2007 criteria, which performed better in Black patients than White patients (area under the curve, 0.873; 95% CI, 0.836-0.907 vs area under the curve, 0.809; 95% CI, 0.788-0.830). All pneumonia severity scores predicted direct ICU admission equally well in White and Black patients. Compared with White patients, Black patients had decreased incidence of severe CAP as defined by all pneumonia severity scores except the Severe Community-Acquired Pneumonia score (11.5% in White patients vs 10.0% in Black patients; *P* = .146).

**Interpretation:**

Our results show that most pneumonia severity scores perform equally well for predicting in-hospital mortality and direct ICU admission in White and Black patients except the ATS/IDSA 2007 criteria, which showed superior performance in Black patients when predicting in-hospital mortality. Before implementation in clinical practice, it is important to consider the limitations of pneumonia severity scores and their subsequent impact on equitable care delivery.

**Take-Home Points**

**Study** **Question:** Does Black race differentially impact the ability of pneumonia severity scoring systems to predict in-hospital mortality or direct ICU admission?

**Results:** All pneumonia severity scores predicted in-hospital mortality equally well in White and Black patients except the American Thoracic Society/Infectious Diseases Society of America (ATS/IDSA) 2007 criteria, which showed superior performance in Black patients compared with White patients. All pneumonia severity scores predicted direct ICU admission equally well in White and Black patients.

**Interpretation:** In this study, most pneumonia severity scores performed equally well for predicting in-hospital mortality and direct ICU admission in White and Black patients except the ATS/IDSA 2007 criteria, which showed superior performance in Black patients when predicting in-hospital mortality.

Community-acquired pneumonia (CAP) is one of the most common reasons for hospitalization and ICU admission in the world.^1^ It is well recognized that racial, ethnic, and socioeconomic disparities have a significant impact on various clinical end points in patients with CAP, such as mortality, length of stay, and need for ICU admission. Over the past decade, several studies have examined the impact of structural racism on the US health care system, which can further exacerbate some of the racial disparities in CAP.^2^, ^3^, ^4^

A complex interplay of social, financial, and structural mechanisms contribute to the racial disparities seen in CAP. Compared with White patients, Black patients are more likely to be hospitalized with CAP and have a higher incidence of bacterial pneumonia even when controlled for age and socioeconomic level.^5^ Additionally, Black patients are less likely to receive their first dose of antibiotic within 4 hours, are less likely to receive pneumococcal/influenza vaccination, are less likely to receive tobacco cessation counseling, and experience higher readmission rates after being discharged from the hospital for CAP.^4^^,^^6^ Although prior reports from the early 2000s suggest that White and Black patients are equally as likely to receive guideline-concordant antibiotic therapy for CAP, a report on patients (after publication of the 2019 American Thoracic Society/Infectious Diseases Society of America [ATS/IDSA] guidelines for management of CAP) in the All of Us database found that Black patients were less likely to receive guideline-concordant antibiotics compared with non-Hispanic White patients.^4^^,^^7^^,^^8^

Various scoring systems are used to compare patient populations, are used to assess treatment response, and are sometimes used to direct patient care. Pneumonia severity scores (eg, Pneumonia Severity Index [PSI], CURB-65) are essential tools in the management of CAP, which help guide clinical decision-making, including the need for hospitalization, level of care, and intensity of treatment. Racial differences in severity of illness exist and scoring systems that assess severity of illness need to be comprehensively evaluated to better recognize potential biases in the models because they may significantly impact the care received by patients and amplify existing racial bias in health care.^9^^,^^10^

Studies have shown racial disparities in the predictive ability of several illness severity scores which were designed to predict risk for various clinical end points (mortality, ICU admission, and hospital length of stay). For example, evidence suggests that the Sequential Organ Failure Assessment (SOFA) overestimates in-hospital mortality in Black patients, which is a disadvantage in Crisis Standards of Care (CSC) algorithms and has led some authors to suggest using a modified SOFA score.^11^^,^^12^ A large multicenter retrospective study (N = 166,751) of ICU patients (before COVID-19) found that the SOFA, Acute Physiology and Chronic Health Evaluation (APACHE IVa), and Oxford Acute Severity of Illness scores consistently overpredicted mortality for Black and Hispanic patients, making the use of these scores for clinical resource allocation across racial and ethnic groups problematic.^13^ These examples illustrate that clinical scoring systems may have inherent risk for bias in their predictive performance due to differences in calibration across race/ethnicity, which need to be accounted for if used in clinical practice for guideline and policy development.

Patients from racial and ethnic minority groups with sepsis-related respiratory failure have worse outcomes than patients who are not from racial and ethnic minority groups, and delay in admission to the ICU in patients with CAP is associated with increased mortality.^14^ Multiple scoring systems have been developed to assist in directing patient care by assessing the severity of CAP and predict mortality and risk for ICU admission. In 2019, the ATS/IDSA published guidelines on the treatment of CAP which strongly encourage the adjunctive use of scoring systems to assist in determining the site of care and to define severe CAP, which has implications on treatment recommendations.^15^

Despite many studies illustrating that Black patients have higher mortality and are at increased risk for ICU admission for respiratory failure, no study has assessed the impact of race on the calibration and discrimination of pneumonia severity models in adults. It is imperative to assess the validity of these scoring systems in patients from racial and ethnic minority populations to ensure that health care resources are allocated appropriately and equitably. The primary aim of this study is to evaluate the ability of pneumonia severity scoring systems to predict in-hospital mortality in White and Black patients with CAP. The secondary aims of this study are to evaluate the predictive ability of pneumonia severity scoring systems to determine severe CAP based on clinical cutoff points and direct ICU admission.

## Study Design and Methods

This is a secondary analysis of 2 prospective cohorts of patients ≥ 18 years of age who were hospitalized with SARS-CoV-2 CAP or non-SARS-CoV-2 CAP from 8 adult hospitals in Louisville, Kentucky.^16^ There were no exclusion criteria. A patient was defined as having CAP when both of the following criteria were met: (1) fever, cough, or shortness of breath; and (2) evidence of pulmonary infiltrates on chest radiography or chest CT scan, as determined by a board-certified radiologist, and obtained within 48 hours of admission. Patients in the SARS-CoV-2 CAP cohort satisfied the previous requirements for the diagnosis of CAP and had a positive reverse transcription polymerase chain reaction for SARS-CoV-2. Patients transferred to hospice were counted as in-hospital deaths. This study was approved by the University of Louisville Institutional Review Board (No. 20.0257), and it was exempt from informed consent.

### Data Collection

Research associates screened patients daily for CAP at all participating hospitals from June 1, 2014, to May 31, 2016 (non-SARS-CoV-2 CAP cohort) and March 5, 2020, to March 31, 2021 (SARS-CoV-2 CAP cohort).^16^^,^^17^ Clinical variables (eg, demographics, comorbidities, physical examination, radiographic data) were collected from hospital admission. Race was determined based on documentation in the medical record. Radiographic pneumonia was identified as an infiltrate by a board-certified radiologist on CT scan or chest radiograph within 48 hours of admission. Vital signs and laboratory data used to calculate pneumonia severity scores represent the most extreme values within the first 24 hours of admission. If blood gases were not obtained, we considered arterial pH > 7.35, thus contributing no points to the PSI calculation, which is consistent with prior analysis.^18^ Additionally, if blood gases were not obtained, we used oxygen saturation < 90% as a surrogate measure for Pao_2_ < 60 mm Hg, consistent with the PSI model.^19^ All data were stored in a Health Insurance Portability and Accountability Act of 1996-protected online platform.

### Severity Scores

The following pneumonia severity scores were assessed for each patient: PSI, CURB-65, Confusion, Oxygenation, Respiratory Rate, and Blood Pressure (CORB) scale, ATS/IDSA 2007 criteria, SMART-COP score, Severe Community-Acquired Pneumonia (SCAP) score, and Risk of Early Admission to ICU (REA ICU) index.^19^, ^20^, ^21^, ^22^, ^23^, ^24^, ^25^ Details regarding how severe CAP was defined for each score and how each score was calculated for this study are listed in e-Appendix 1. Clinical outcomes assessed in this analysis were in-hospital mortality (all cause) and direct ICU admission (direct admission from the emergency department).

### Statistical Analysis

Continuous data are expressed as median values, and comparison between groups was assessed by the Mann-Whitney *U* test. Categorical variables are expressed as a number (%) and compared by Fisher exact tests or χ^2^ tests of independence, as appropriate. Incidence of severe CAP and severe CAP mortality were described by race for each severity score. Sensitivity, specificity, positive predictive value, and negative predictive value were also stratified by race for each severity score and further stratified by etiology. The 95% CIs were reported for these statistics using the Clopper-Pearson method. Additionally, receiver operator characteristic (ROC) curves and analysis was performed to assess the predictive value of each score for clinical outcomes using the area under the curve (AUC). ROC curves stratified by patient race were also created to analyze the differences in predictive value. ROC analysis was performed for the overall population and further stratified by etiology. Bootstrap 95% CIs for the AUC were calculated using nonparametric stratified resampling with 2,000 samples. *P* < .05 was considered statistically significant. Analysis was performed with R software version 4.3 (R Core Team).

## Results

### Baseline Characteristics

A total of 9,797 patients were hospitalized for CAP (8,283 with non-SARS-CoV-2 CAP and 1,513 with SARS-CoV-2 CAP). A schematic for this study is shown in Figure 1 (e-Table 1). There were 6,479 White patients with a median age of 69 years (interquartile range, 58-81), and 1,780 Black patients with a median age of 61 years (interquartile range, 50-73). Baseline characteristics are summarized in Table 1. Notably, compared with White patients, Black patients had an increased incidence of diabetes (44.8% vs 30.8%, *P* < .001), heart failure (30.1% vs 25.8%, *P* < .001), and renal disease (33.8% vs 26.7%, *P* < .001). Although the percentage of Black and White patients requiring ICU admission during hospitalization was similar (23.9% vs 23.6%, *P* = .819), Black patients were more likely to require transfer to the ICU during hospitalization (8.7% vs 6.6%, *P* = .003). When broken down by PSI risk class or CURB-65 score, mortality was similar between White and Black patients within each risk class; however, at higher severity (PSI > 4 or CURB-65 > 3), mortality was higher in White patients (e-Fig 1). On discharge, death had occurred in 5.6% of Black patients and 7.5% of White patients (*P* = .007). Compared with White patients, Black patients had a higher incidence of SARS-CoV-2 pneumonia, lower incidence of bacterial pneumonia, and lower incidence of CAP with no specific etiology detected (e-Table 2). In-hospital mortality and rate of ICU admission grouped by etiology and race are listed in e-Table 3.

**Figure 1:**
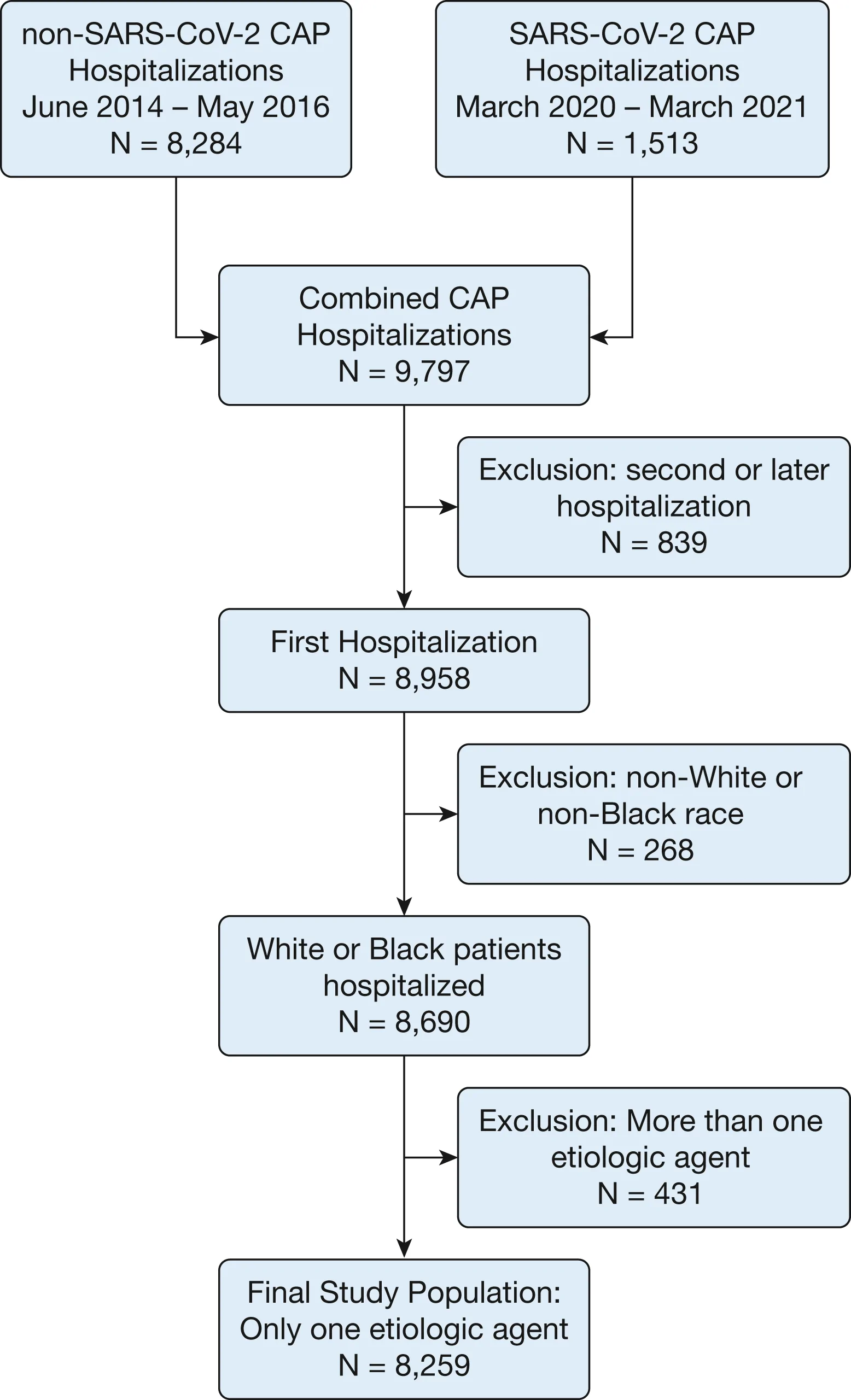
Study flowchart of cohort selection criteria.

**Table 1:** Baseline Characteristics and Laboratory Values for Patients With Community-Acquired Pneumonia

Variable	White Patients (n = 6,479)	Black Patients (n = 1,780)	*P* Value
Age, y	69 (58-81)	61 (50-73)	< .001
Sex, male	3,065 (47.3)	772 (43.4)	.003
Nursing home resident	848 (13.1)	219 (12.3)	.404
BMI, kg/m^2^	27.1 (22.8-32.5)	29.8 (23.9-36.4)	< .001
Vitals			
Heart rate	104 (89-118)	104 (92-117)	.510
SBP < 90 mm Hg or DBP < 60 mm Hg	3,779 (58.3)	798 (44.9)	< .001
Respiratory rate ≥ 30 breathes/min	1,081 (16.7)	311 (17.5)	.442
Temperature < 95 °F (35 °C)	40 (0.6)	9 (0.5)	.714
Temperature ≥ 100.4 °F (38 °C)	1,547 (23.9)	464 (26.1)	.057
Medical history			
Any comorbidity	5,578 (96.0)	1,431 (96.2)	.787
> 2 comorbidities	4,849 (83.4)	1,273 (85.6)	.052
COPD	2,865 (44.2)	652 (36.6)	< .001
Diabetes	1,995 (30.8)	798 (44.8)	< .001
Heart failure	1,672 (25.8)	535 (30.1)	< .001
Hypertension	4,372 (67.5)	1,339 (75.2)	< .001
Stroke	810 (12.5)	242 (13.6)	.236
Neoplastic disease within past year	863 (13.3)	190 (10.7)	.003
Renal disease	1,732 (26.7)	602 (33.8)	< .001
Liver disease	420 (6.5)	117 (6.6)	.934
Currently smokes	1,773 (27.4)	571 (32.1)	< .001
Formerly smoked	2,509 (38.7)	553 (31.1)	< .001
Laboratory results on admission			
Lymphocyte count, cells/mm^3^	11.9 (8.1-16.2)	9.7 (6.6-14.3)	< .001
Platelets, cells/mm^3^	219 (166-284)	221 (172-286)	.119
Hematocrit	36.2 (31.9-40.3)	35.6 (31.3-39.9)	.004
Albumin, g/dL	3.6 (3.1-4.0)	3.6 (3.2-4.0)	.012
Creatinine, mg/dL	1.0 (0.8-1.4)	1.1 (0.8-1.8)	< .001
Sodium, mmol/L	136 (134-140)	138 (135-141)	< .001
CRP, mg/L^a^	21.8 (7.0-73.7)	22.0 (8.0-79.3)	.443
Procalcitonin, ng/mL^b^	0.14 (0.05-0.73)	0.22 (0.06-1.23)	.002
pH^c^	7.41 (7.35-7.45)	7.40 (7.34-7.44)	.052
Pao_2_/Fio_2_^c^			
> 300	524 [20.7]	170 [26.0]	.005
200-300	886 [34.3	197 [30.]	.048
100-199	787 [31.1]	179 [27.3]	.066
< 100	351 [13.9]	109 [16.6]	.084
Clinical variables			
Altered mental status	1,211 (18.7)	276 (15.5)	.002
Pleural effusion	2,008 (31.0)	482 (27.1)	.002
ICU admission	1,529 (23.6)	426 (23.9)	.819
Direct ICU admission	1,101 (17.0)	272 (15.2)	.082
ICU transfer	428 (6.6)	154 (8.7)	.003
Invasive mechanical ventilation on admission	368 (5.7)	106 (6.0)	.701
Vasopressors on admission	201 (3.1)	57 (3.2)	.890
Death at discharge	486 (7.5)	100 (5.6)	.007

Data are presented as No. (%), mean [SD], median (interquartile range), or as otherwise indicated. CRP = C-reactive protein; DBP = diastolic BP; SBP = systolic BP.

a CRP was taken in 703 White and 280 Black patients.

b Procalcitonin was taken in 1,998 White and 540 Black patients.

c Arterial blood gasses were obtained in 2,528 White and 655 Black patients.

### Predictive Value for In-Hospital Mortality

All pneumonia severity scoring systems were able to predict in-hospital mortality with an AUC > 0.674 (Fig 2, Table 2). Among all patients, the least predictive scoring systems for in-hospital mortality were the CORB, SMART-COP, SCAP, and REA ICU scores with AUC values of 0.710, 0.701, 0.733, and 0.688, respectively. Within the CORB, SMART-COP, SCAP, and REA ICU scoring systems, there was no statistically significant difference in performance between White and Black patients. The ATS/IDSA 2007 model performed significantly better in Black patients (AUC, 0.873; 95% CI, 0.836-0.907) than White patients (AUC, 0.809; 95% CI, 0.788-0.830) (e-Table 4). Distinct differences in minor criteria were observed between White and Black patients (e-Table 5). Subgroup analysis by CAP etiology showed that all models predicted in-hospital mortality similarly in both White and Black patients regardless of etiology (e-Figs 2-8, e-Table 6).

**Figure 2:**
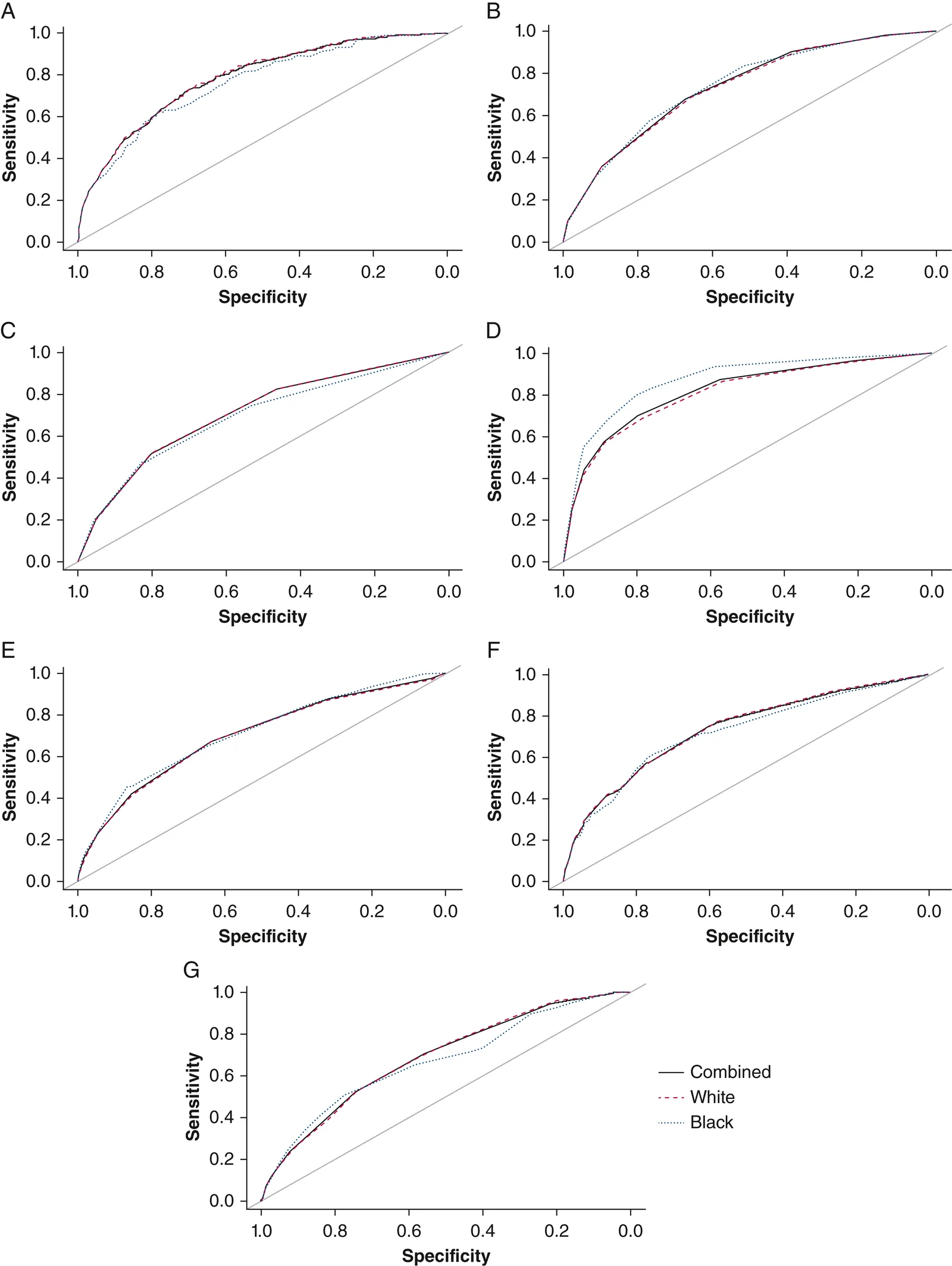
A-G, Receiver operator characteristic plots for in-hospital mortality in White and Black patients by pneumonia severity scoring system: (A) Pneumonia Severity Index; (B) CURB-65; (C) Confusion, Oxygenation, Respiratory Rate, and Blood Pressure scale; (D) American Thoracic Society/Infectious Diseases Society of America 2007 criteria; (E) SMART-COP; (F) Severe Community-Acquired Pneumonia; and (G) Risk of Early Admission to ICU.

**Table 2:** Receiver Operator Characteristic Analysis by Race for Risk of In-Hospital Mortality From Community-Acquired Pneumonia

Severity Score	Race	Sens	Spec	PPV	NPV	AUC (95% CI).
PSI	Combined	0.981	0.108	0.436	0.891	0.783 (0.763-0.801)
	White	0.981	0.111	0.415	0.903	0.786 (0.765-0.806)
	Black	0.980	0.091	0.508	0.830	0.762 (0.711-0.806)
CURB-65	Combined	0.965	0.137	0.676	0.674	0.735 (0.715-0.754)
	White	0.963	0.138	0.651	0.693	0.731 (0.708-0.752)
	Black	0.969	0.128	0.768	0.582	0.741 (0.690-0.785)
CORB	Combined	0.940	0.242	0.952	0.201	0.710 (0.688-0.731)
	White	0.936	0.247	0.950	0.202	0.711 (0.687-0.734)
	Black	0.954	0.221	0.960	0.196	0.694 (0.636-0.747)
ATS/IDSA 2007 criteria	Combined	0.984	0.137	0.575	0.881	0.821 (0.803-0.839)
	White	0.982	0.140	0.568	0.870	**0.809 (0.788-0.830)**
	Black	0.993	0.121	0.599	0.930	**0.873 (0.836-0.907)**
SMART-COP	Combined	0.951	0.181	0.853	0.426	0.701 (0.678-0.723)
	White	0.948	0.183	0.848	0.422	0.698 (0.671-0.722)
	Black	0.964	0.169	0.871	0.444	0.712 (0.654-0.761)
SCAP	Combined	0.949	0.233	0.910	0.362	0.733 (0.710-0.754)
	White	0.947	0.246	0.909	0.369	0.734 (0.711-0.759)
	Black	0.959	0.181	0.914	0.327	0.720 (0.666-0.771)
REA ICU	Combined	0.962	0.108	0.556	0.707	0.688 (0.665-0.709)
	White	0.960	0.114	0.546	0.719	0.688 (0.665-0.711)
	Black	0.967	0.084	0.591	0.649	0.674 (0.618-0.731)

ATS/IDSA = American Thoracic Society/Infectious Diseases Society of America; AUC = area under the curve; Bold Font = Statistical significance; CORB = Confusion, Oxygenation, Respiratory Rate, and Blood Pressure; NPV = negative predictive value; PPV = positive predictive value; PSI = Pneumonia Severity Index; REA ICU = Risk of Early Admission to ICU; SCAP = Severe Community-Acquired Pneumonia; Sens = sensitivity; Spec = specificity.

### Predictive Value for ICU Admission

Among all patients, the PSI, CURB-65, CORB, ATS/IDSA 2007 criteria, SMART-COP, SCAP, and REA ICU scoring systems were able to predict direct ICU admission with an AUC of 0.774, 0.725, 0.770, 0.848, 0.781, 0.807, and 0.754 (Fig 3, Table 3). Within each scoring system, there were no statistically significant differences in the predictive ability of the scoring systems to predict direct ICU admission in White and Black patients (e-Table 7). Subgroup analysis by CAP etiology showed that all models predicted direct ICU admission similarly in both White and Black patients regardless of etiology (e-Figs 9-15, e-Table 8).

**Figure 3:**
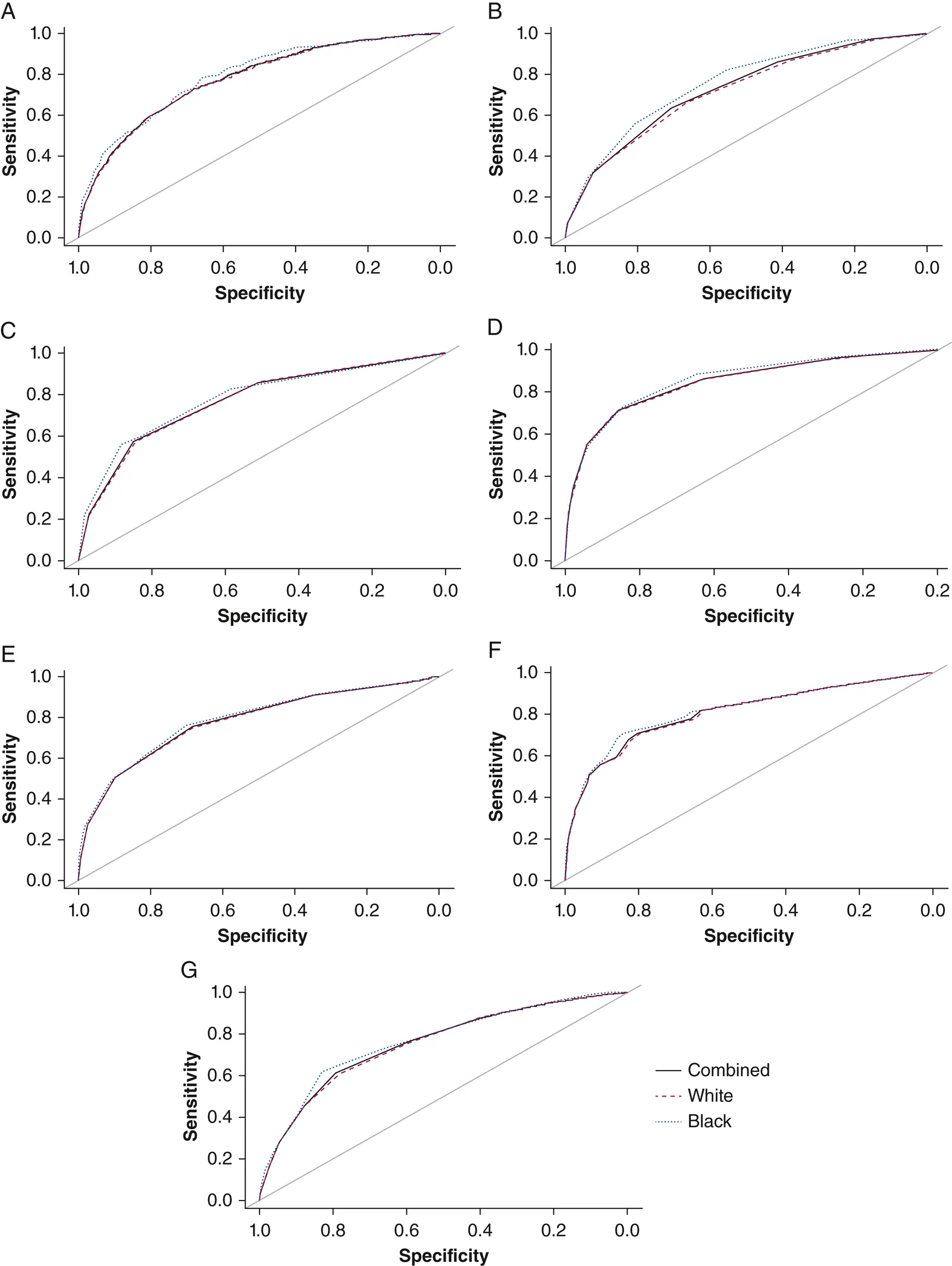
A-G, Receiver operator characteristic plots for direct ICU admission in White and Black patients by pneumonia severity scoring system: (A) Pneumonia Severity Index; (B) CURB-65; (C) Confusion, Oxygenation, Respiratory Rate, and Blood Pressure scale; (D) American Thoracic Society/Infectious Diseases Society of America 2007 criteria; (E) SMART-COP; (F) Severe Community-Acquired Pneumonia; and (G) Risk of Early Admission to ICU.

**Table 3:** Receiver Operator Characteristic Analysis for Risk of Direct ICU Admission From Community-Acquired Pneumonia

Severity Score	Race	Sens	Spec	PPV	NPV	AUC
PSI	Combined	0.946	0.245	0.468	0.867	0.774 (0.761-0.788)
	White	0.944	0.243	0.445	0.872	0.770 (0.755-0.785)
	Black	0.952	0.252	0.549	0.845	0.789 (0.759-0.818)
CURB-65	Combined	0.906	0.301	0.708	0.631	0.725 (0.710-0.740)
	White	0.904	0.293	0.681	0.648	0.717 (0.701-0.733)
	Black	0.911	0.341	0.805	0.563	0.756 (0.725-0.784)
CORB	Combined	0.863	0.625	0.974	0.221	0.770 (0.757-0.784)
	White	0.859	0.607	0.971	0.219	0.767 (0.751-0.782)
	Black	0.877	0.709	0.983	0.227	0.781 (0.752-0.812)
ATS/IDSA 2007 criteria	Combined	0.960	0.316	0.625	0.870	0.848 (0.836-0.859)
	White	0.957	0.316	0.617	0.866	0.846 (0.831-0.859)
	Black	0.969	0.313	0.651	0.886	0.858 (0.831-0.882)
SMART-COP	Combined	0.901	0.503	0.900	0.505	0.781 (0.767-0.795)
	White	0.900	0.504	0.897	0.511	0.780 (0.764-0.795)
	Black	0.908	0.498	0.913	0.481	0.787 (0.756-0.816)
SCAP	Combined	0.891	0.633	0.952	0.418	0.807 (0.793-0.820)
	White	0.889	0.635	0.951	0.420	0.805 (0.789-0.821)
	Black	0.900	0.621	0.955	0.407	0.816 (0.783-0.846)
REA ICU	Combined	0.926	0.273	0.597	0.761	0.754 (0.741-0.768)
	White	0.924	0.274	0.586	0.765	0.751 (0.735-0.767)
	Black	0.934	0.268	0.636	0.747	0.765 (0.732-0.795)

ATS/IDSA = American Thoracic Society/Infectious Diseases Society of America; AUC = area under the curve; CORB = Confusion, Oxygenation, Respiratory Rate, and Blood Pressure; NPV = negative predictive value; PPV = positive predictive value; PSI = Pneumonia Severity Index; REA ICU = Risk of Early Admission to ICU; SCAP = Severe Community-Acquired Pneumonia; Sens = sensitivity; Spec = specificity.

### Predictive Value for Severe CAP Based on Clinical Cutoffs

Commonly accepted clinical cutoff points (e-Appendix 1) that define severe CAP for each pneumonia severity model were used to assess the incidence of severe CAP in this cohort. There was a wide range of severe CAP in White (6.1%-60.9%) and Black (4.9%-51.1%) patients. Within most scoring systems, differences in the incidence of severe CAP between White and Black patients was within approximately 5%, but notably the difference was greatest in the CURB-65 (37.5% in White patients and 25.1% in Black patients) and PSI models (60.9% in White patients and 51.1% in Black patients). All models, except SCAP, showed a significantly higher incidence of severe CAP in White patients than Black patients (Table 4). When the in-hospital mortality was assessed for patients with severe CAP, there was no significant difference by race except for patients identified by the REA ICU index; Black patients experienced lower in-hospital mortality (8.4%) than White patients (11.4%) (*P* = .024).

**Table 4:** Severe Community-Acquired Pneumonia Based on Clinical Cutoffs

Severity Score	Race	Severe CAP Incidence	*P* Value	Severe CAP Mortality^a^	*P* Value
PSI	White (n = 6,479)	3,943 (60.9)	< .001	439 (11.1)	.090
	Black (n = 1,780)	909 (51.1)		83 (9.1)	
CURB-65	White (n = 6,479)	2,430 (37.5)	< .001	336 (13.8)	.605
	Black (n = 1,780)	446 (25.1)		57 (12.8)	
CORB	White (n = 6,479)	397 (6.1)	.047	98 (24.7)	.712
	Black (n = 1,780)	86 (4.9)		19 (22.1)	
ATS/IDSA 2007 criteria	White (n = 6,479)	3,012 (46.5)	.010	423 (14.0)	.190
	Black (n = 1,780)	766 (43.0)		93 (12.1)	
SMART-COP	White (n = 6,479)	1,118 (17.3)	.011	205 (18.3)	.639
	Black (n = 1,780)	261 (14.7)		44 (16.9)	
SCAP	White (n = 6,479)	727 (11.2)	.146	179 (24.6)	.081
	Black (n = 1,780)	177 (10.0)		32 (18.1)	
REA ICU	White (n = 6,479)	3,066 (47.4)	< .001	348 (11.4)	.024
	Black (n = 1,780)	749 (42.2)		63 (8.4)	

Data are presented as No. (%) or as otherwise indicated. ATS/IDSA = American Thoracic Society/Infectious Diseases Society of America; CAP = community-acquired pneumonia; PSI = Pneumonia Severity Index; REA ICU = Risk of Early Admission to ICU; SCAP = Severe Community-Acquired Pneumonia.

a Severe CAP mortality is the number of patients with severe CAP, as defined by each severity score, that experienced in-hospital mortality.

## Discussion

This study assessed the impact of race on the ability of pneumonia severity scoring systems to predict in-hospital mortality, direct ICU admission from the emergency department, and identify severe CAP. There was a wide range in the discrimination of different pneumonia severity scoring systems to predict in-hospital mortality for all patients regardless of race (AUC, 0.688 [REA ICU] to AUC, 0.821 [ATS/IDSA 2007 criteria]). When predicting in-hospital mortality, the ATS/IDSA 2007 criteria showed significantly improved performance in Black patients (AUC 0.873; 95% CI, 0.836-0.907) compared with White patients (AUC, 0.809; 95% CI, 0.788-0.830), whereas all other pneumonia severity scoring systems showed no significant difference based on race. Similarly, there was a wide range in the ability of pneumonia severity scoring systems to predict direct ICU admission (AUC, 0.770 [CORB] to AUC, 0.848 [ATS/IDSA 2007 criteria]), with no significant difference in performance between White and Black patients. In this cohort, Black patients presented with less severe pneumonia than White patients based on commonly accepted clinical cutoff points for PSI, CURB-65, CORB, ATS/IDSA 2007 guidelines, SMART-COP, and the REA ICU index, and overall mortality was lower in Black patients (5.6%) than White patients (7.5%). Subgroup analysis of in-hospital mortality and direct ICU admission by CAP etiology revealed no significant differences between White and Black patients in the predictive capacity of PSI, CURB-65, CORB, ATS/IDSA 2007 criteria, SMART-COP, SCAP, and REA ICU. When in-hospital mortality for patients was assessed for severe CAP as defined by different pneumonia severity scores, Black patients had lower mortality than White patients (11.4% vs 8.4%; *P* = .024) as determined by the REA ICU index, whereas all other pneumonia severity scoring systems showed no significant difference based on race. To our knowledge, this is the first study to comprehensively evaluate the impact of race on major pneumonia severity scoring systems that have been developed over the past several decades. Our results indicate that there is wide variability in the predictive capacity of pneumonia severity scoring systems.

Multiple large studies have illustrated the disproportionate morbidity and mortality of CAP among Black US adults compared with White US adults. For example, in the 1990s, 1 study showed the incidence of CAP to be higher in Black adults than White adults (337.7 per 100,000 vs 253.9 per 100,000, respectively).^26^ In the early 2000s, Burton et al^5^ showed that the annual incidence of bacterial pneumonia in Black adults was 24.2 per 100,000 vs 10.1 per 100,000 in White adults. A report on mortality from influenza and pneumonia from 1999 to 2018 found that age-adjusted mortality rates were higher in non-Hispanic Blacks than non-Hispanic Whites in every age group from 25-84 years of age in the US, and regions of the country with the greatest racial disparities in mortality were also hotspots for SARS-CoV-2 pneumonia.^27^ There are wide variations in racial disparities in different regions of the US, and evidence suggests that there are underlying systemic issues that contribute to these disparities.^4^^,^^6^ Despite there being much interest in the use of models to assess illness severity in pneumonia so that more aggressive care and resources can be provided to those with severe CAP, caution must be exercised when applying scoring systems to individual patients to predict outcomes, in part because the CIs surrounding scoring systems are broad, resulting in frequent misclassification of patients.^28^

There has been much debate in the literature about the use of race-specific models in medicine which has resulted in a shift toward the use of race-neutral models (models which do not explicitly incorporate race); however, there is a relative paucity of data on the impact of race on race-neutral models that are used in medical practice.^29^ Regardless, the use of race correction to guide care in clinical algorithms comes at great risk. Models that do not incorporate race as a factor can still exhibit racial bias if they were not developed and calibrated in a representative homogenous population. For example, scores which are commonly used to assess illness severity in critical care such as SOFA, APACHE IVa, and Oxforda Acute Severity of Illness have been shown to overpredict mortality in Black and Hispanic patients.^11^, ^12^, ^13^ Riviello et al^30^ assessed a CSC algorithm (composed of the SOFA score, comorbidities, and life expectancy) that was originally developed in Massachusetts to assist in the allocation of scarce medical resources during the COVID-19 pandemic; although it was never used, they found that Black patients were more likely to be in the lowest priority group. In another study, there was no association with race or ethnicity in a CSC algorithm developed in Florida, highlighting the variability of CSC algorithms and raising the importance of using models that have been validated across different populations before implementation.^31^ In CSC algorithms, having a higher predicted mortality rate based on race may adversely impact the receipt of medical care in resource-poor settings.

The ATS/IDSA 2007 criteria for severe CAP is 1 of the most accepted criteria to define severe CAP. Some clinical trials investigating treatment for severe CAP have used the ATS/IDSA 2007 criteria as inclusion criteria, which may be problematic if the expected mortality is different for White vs Black patients as illustrated by our data. For example, several studies investigating the effects of corticosteroids in severe CAP have used the ATS/IDSA 2007 criteria (or a modified version) as part of their inclusion criteria.^32^, ^33^, ^34^ Additionally, retrospective studies in patients with severe CAP have also used the ATS/IDSA 2007 criteria to define severe CAP.^35^ Overall, 87% of White patients and 93% of Black patients who died in the cohort in our study were identified as severe CAP by ATS/IDSA 2007 criteria, suggesting that these criteria can continue to be used to identify patients at high risk for in-hospital mortality. Prior studies have shown that the individual ATS/IDSA 2007 minor criteria contribute unequally to the prediction of mortality and ICU admission.^36^, ^37^, ^38^, ^39^ In our study, the proportion of White and Black patients meeting major criteria (mechanical ventilation or vasopressor requirement) was similar. However, the distribution of fulfilled minor criteria differed between groups. These differences may underlie the variable performance of the ATS/IDSA 2007 criteria in predicting in-hospital mortality across racial groups.

There are limitations in our study. Although this study is multicenter, it is limited to a single city in the US which can affect generalizability. Additionally, race was recorded based on documentation in the medical record. It is possible that race was not self-reported but determined by clinical staff at participating hospitals, which may impact the accuracy of our data. Although blood gases were not obtained on all patients, sensitivity analysis on the subset of patients with arterial blood gases confirmed our original findings. Our study has several strengths. Our database is 1 of the largest CAP databases in the US, which includes clinical variables that were prospectively collected and validated by research associates. Additionally, prior work has shown that the population of Louisville, Kentucky, is highly representative of the US in terms of health-related statistics, demographics, and socioeconomic factors.^16^^,^^40^ Consistent with prior work, our work showed a similar rate of White and Black patients requiring ICU admission throughout hospitalization; however, compared with White patients, more Black patients required transfer to the ICU after admission and there was a trend toward fewer Black patients directly admitted to the ICU (Table 1).^41^ Although our study shows improved mortality in Black patients compared with White patients, which is consistent with several prior reports, there are several notable studies which have showed higher adjusted mortality rates in Black patients, illustrating the complexity of assessing health care racial disparities.^3^^,^^4^^,^^6^^,^^7^^,^^27^^,^^42^ Consistent with previous studies, we assessed the predictive performance of multiple pneumonia severity scoring systems across a range of clinical outcomes. Notably, evaluation of in-hospital mortality by the ATS/IDSA 2007 criteria has not been previously validated, which underscores the need for additional validation studies to determine whether the ATS/IDSA 2007 criteria perform equitably across different racial groups.

## Interpretation

Although multiple studies suggest that racial disparities persist in universal health care insurance programs (Medicare, Medicaid, Veteran’s Affairs, and the US Military Health System), evidence shows that some disparities are improved when quality and access to care are similar between White and Black patients.^43^, ^44^, ^45^, ^46^ Except for the ATS/IDSA 2007 criteria, which performed better in Black patients for the prediction of in-hospital mortality, there was no statistical difference for all other models in the prediction of either in-hospital mortality or ICU admission. In conclusion, our data show that Black race does not influence most illness severity models in CAP to predict in-hospital mortality and ICU admission.

## Funding/Support

This paper was supported by The Center for Excellence for Research in Infectious Diseases at the University of Louisville.

## Financial/Nonfinancial Disclosures

None declared.

## Supplementary Data

e-Online Data

e-Online Data

e-Online Data

e-Online Data

e-Online Data

e-Online Data

e-Online Data

e-Online Data

e-Online Data

e-Online Data

e-Online Data

e-Online Data

e-Online Data

e-Online Data

e-Online Data

e-Online Data

e-Online Data
