# Supplementary material for: The Impact of Black Race on Pneumonia Severity Scoring Systems
Source: CHEST Pulm. 2026 Feb 5;4(3):100234. doi: 10.1016/j.chpulm.2026.100234 (PMC13617195; doi:10.1016/j.chpulm.2026.100234)
Supplement: Supplementary file 1 [file mmc1.docx]

**Supplementary Methods – Pneumonia Severity Models**

**Pneumonia Severity Index (PSI)**

Pneumonia Severity Index (PSI) is a prediction rule that was originally designed to identify patients with CAP who are at low risk of death and other adverse outcomes.^1^ PSI is a point-based severity score where points are awarded for clinically relevant pre-existing conditions, initial exam findings, and lab values. For ROC plotting and AUC calculations the point total for each patient was used. PSI is broken into risk classes I-V. The clinical cutoff for severe CAP to predict in-hospitality mortality and ICU admission was PSI risk class of IV or V.

**CURB-65 / CORB**

CURB-65 is a six-point score based on confusion, urea, respiratory rate, blood pressure and age.^2^ A patient gets a point for each of the criteria being met and receives zero points if the criteria is not met. This prediction rule was originally designed with 30-day mortality as the outcome measure. For completeness we have also considered a slight variation of CURB-65 called CORB, where O is oxygen saturation.^3^ For ROC plotting and AUC calculations, the total points for each was used. The clinical cutoff for severe CAP in predicting in-hospital mortality and ICU admission was a score of 3-5 for CURB-65 and a score of 3-4 for CORB.

**ATS/IDSA 2007**

In 2007,^,^ the Infectious Diseases Society of America (IDSA) and American Thoracic Society (ATS) released guidelines related to hospital admissions and ICU admission decisions.^4^ The clinical cutoff for direct ICU admission was recommended for patients with three of the minor criteria or one of two major criteria. This clinical cutoff was used to predict in-hospital mortality and ICU admission among patients in our model. For ROC plotting and AUC calculations, two points were awarded for each major criterion and one point for each minor criterion to create a score for analysis. The clinical cutoff for severe CAP to predict in-hospital mortality and ICU admission was the presence of one or more major criterion and/or the presence of three or more minor criterion.

**SMART COP**

SMART COP is a predictive pneumonia severity score created for predicting the need for intensive respiratory or vasopressor support for patients with CAP.^5^ SMART COP is a scoring system with a range from 0-11 points, and the clinical cutoff of ≥ 5 points represents a high risk of needing intensive respiratory or vasopressor support. This clinical cutoff was used in predicting in-hospital mortality and ICU admission among patients in our model. For ROC plotting and AUC calculations, the raw score from the scoring system was used. The clinical cutoff for severe CAP to predict in-hospital mortality and ICU admission was 5 or more points.

**SCAP**

Severe Community Acquired Pneumonia (SCAP) is a score created for predicting severe CAP and it was originally derived from patients evaluated in the emergency room.^6^ The score consists of eight clinical variables with various points associated with each variable. The score range is 0 – 59 points, and these scores are then stratified by intervals of 10 points to create risk classes. No clinical cutoff for severe CAP is suggested from the original publication. The clinical cutoff used in this analysis for severe CAP to predict in-hospital mortality and ICU admission was a score of 20 points or higher. For ROC plotting and AUC calculations, the raw score from the scoring system was used.

**REA ICU**

Risk of Early Admission to Intensive Care Unit (REA ICU) is a model designed to predict the need for ICU admission within three days of presentation to the emergency department.^7^ This score includes various points for 11 clinical, laboratory and x-ray parameters. The score range is 0-17 points, and the scores are then separated into risk classes I-IV. Risk classes III and IV were considered the cut point for need for early ICU admission in the original score. This clinical cut point was used for predicting in-hospital mortality and ICU admission among patients in our study. For ROC plotting and AUC calculations, the raw score from the scoring system was used. The clinical cutoff for severe CAP in predicting in-hospital mortality and ICU admission was seven or more points.

**References**

1.	Fine MJ, Auble TE, Yealy DM, et al. A prediction rule to identify low-risk patients with community-acquired pneumonia. *N Engl J Med.* 1997;336(4):243-250.

2.	Lim WS, van der Eerden MM, Laing R, et al. Defining community acquired pneumonia severity on presentation to hospital: an international derivation and validation study. *Thorax.* 2003;58(5):377-382.

3.	Buising KL, Thursky KA, Black JF, et al. Identifying severe community-acquired pneumonia in the emergency department: a simple clinical prediction tool. *Emerg Med Australas.* 2007;19(5):418-426.

4.	Mandell LA, Wunderink RG, Anzueto A, et al. Infectious Diseases Society of America/American Thoracic Society consensus guidelines on the management of community-acquired pneumonia in adults. *Clin Infect Dis.* 2007;44 Suppl 2(Suppl 2):S27-72.

5.	Charles PG, Wolfe R, Whitby M, et al. SMART-COP: a tool for predicting the need for intensive respiratory or vasopressor support in community-acquired pneumonia. *Clin Infect Dis.* 2008;47(3):375-384.

6.	España PP, Capelastegui A, Gorordo I, et al. Development and validation of a clinical prediction rule for severe community-acquired pneumonia. *Am J Respir Crit Care Med.* 2006;174(11):1249-1256.

7.	Labarère J, Schuetz P, Renaud B, Claessens YE, Albrich W, Mueller B. Validation of a clinical prediction model for early admission to the intensive care unit of patients with pneumonia. *Acad Emerg Med.* 2012;19(9):993-1003.
