# Supplementary material for: The Impact of Black Race on Pneumonia Severity Scoring Systems
Source: CHEST Pulm. 2026 Feb 5;4(3):100234. doi: 10.1016/j.chpulm.2026.100234 (PMC13617195; doi:10.1016/j.chpulm.2026.100234)
Supplement: Supplementary file 2 [file mmc10.docx]

**e-Table 1.** Etiological agents of community-acquired pneumonia.

| Etiology of CAP | N |
| --- | --- |
| Total | 8,259 |
| Non-COVID-19, no organism identified | 5,595 |
| Bacterial | 1,105 |
| Acinetobacter spp. | 10 |
| Actinomyces pp. | 2 |
| Bacteroides spp. | 4 |
| Chlamydia pneumonia | 1 |
| Citrobacter spp. | 2 |
| Enterobacter spp. | 13 |
| Escherichia coli | 23 |
| Haemophilus influenzae | 51 |
| Klebsiella pneumonia | 25 |
| Legionella spp. | 8 |
| Moraxella catarrhalis | 11 |
| Morganella spp. | 1 |
| MRSA | 128 |
| MSSA | 76 |
| Mycoplasma pneumonia | 15 |
| Non-tuberculosis mycobacterium | 1 |
| Pasturella multocida | 1 |
| Proteus spp. | 7 |
| Pseudomonas aeruginosa | 51 |
| Pseudomonas non-aeruginosa | 2 |
| Salmonella spp. | 2 |
| Serratia spp. | 6 |
| Staphylococcus other | 43 |
| Stenotrophomonas spp. | 3 |
| Streptococcus other | 31 |
| Streptococcus pneumonia | 573 |
| Streptococcus pyogenes | 15 |
| Viral | 1,487 |
| Adenovirus | 8 |
| Coronavirus 2019 | 1,153 |
| Coronavirus 229E | 2 |
| Coronavirus HKU1 | 6 |
| Coronavirus NL63 | 2 |
| Coronavirus OC43 | 8 |
| Influenza A 2009 H1N1 | 18 |
| Influenza A H1 | 5 |
| Influenza A H3 | 14 |
| Influenza A untyped | 66 |
| Influenza B | 20 |
| Metapneumovirus | 45 |
| Parainfluenza virus 1 | 4 |
| Parainfluenza virus 2 | 1 |
| Parainfluenza virus 3 | 12 |
| Parainfluenza virus 4 | 4 |
| Respiratory syncytial virus A | 6 |
| Respiratory syncytial virus, unknown type | 23 |
| Rhinovirus/Enterovirus | 90 |
| Fungal | 9 |
| Aspergillus | 8 |
| Pneumocystis jirovecii | 1 |
| Other | 63 |

**e-Table 2. Etiology of community-acquired pneumonia and incidence by race.**

PNA, pneumonia.

| Pneumonia Etiology | Race | Incidence (n, %) | p-value |
| --- | --- | --- | --- |
| Other (unidentified) | White | 4,482 (69.2%) | <0.001 |
|  | Black | 1,185 (66.5%) |  |
|  |  |  |  |
| Bacterial PNA | White | 924 (14.3%) | <0.001 |
|  | Black | 181 (10.2%) |  |
|  |  |  |  |
| Non-SARS-CoV-2 Viral PNA | White | 273 (4.2%) | 0.154 |
|  | Black | 61 (3.4%) |  |
|  |  |  |  |
| SARS-CoV-2 PNA | White | 800 (12.3%) | <0.001 |
|  | Black | 353 (19.8%) |  |

**e-Table 3. In-hospital mortality and direct ICU admission rate by race and etiology for community-acquired pneumonia.**

PNA, pneumonia.

| Etiology | Race | In-hospital Mortality (n, %) | Direct ICU Admission (n, %) |
| --- | --- | --- | --- |
| Overall | Combined | 586 (7.10%) | 1,372 (16.61%) |
| (n=8,259) | White | 486 (7.50%) | 1,101 (16.99%) |
|  | Black | 100 (5.62%) | 271 (15.22%) |
|  |  |  |  |
| Bacterial PNA | Combined | 75 (6.79%) | 286 (25.88%) |
| (n=1,105) | White | 60 (6.49%) | 240 (25.97%) |
|  | Black | 15 (8.29%) | 46 (25.41%) |
|  |  |  |  |
| Non-SARS-CoV-2 Viral PNA | Combined | 12 (3.59%) | 41 (12.28%) |
| (n=334) | White | 12 (4.40%) | 34 (12.45%) |
|  | Black | 0 (0%) | 7 (11.48%) |
|  |  |  |  |
| SARS-CoV-2 PNA | Combined | 180 (15.61%) | 190 (16.48%) |
| (n=1,153) | White | 135 (16.88%) | 144 (18.00%) |
|  | Black | 45 (12.75%) | 46 (13.03%) |

**e-Table 4. Sensitivity Analysis: ROC Analysis by race for risk of in-hospital mortality from community-acquired pneumonia, patient subset with arterial blood gases obtained (n=3183)**

PSI: Pneumonia severity index; CURB-65: Confusion, urea > 7 mmol/L, respiratory rate ≥ 30 breaths/min, blood pressure < 90 (systolic) or < 60 mmHg (diastolic), age ≥ 65; PNA: pneumonia; Sens: sensitivity; Spec, specificity; PPV, positive predictive value; NPV, negative predictive value; AUC: area under the curve; PNA, pneumonia.

| Severity Score | Race | Sens | Spec | PPV | NPV | AUC |
| --- | --- | --- | --- | --- | --- | --- |
| PSI | Combined | 0.970 | 0.163 | 0.309 | 0.933 | 0.738 (0.712-0.763) |
|  | White | 0.964 | 0.172 | 0.294 | 0.930 | 0.731 (0.702-0.760) |
|  | Black | 0.986 | 0.128 | 0.364 | 0.949 | 0.768 (0.704-0.828) |
|  |  |  |  |  |  |  |
| CURB-65 | Combined | 0.932 | 0.194 | 0.575 | 0.710 | 0.698 (0.672-0.723) |
|  | White | 0.927 | 0.201 | 0.549 | 0.724 | 0.690 (0.660-0.719) |
|  | Black | 0.948 | 0.157 | 0.669 | 0.627 | 0.725 (0.662-0.779) |
|  |  |  |  |  |  |  |
| CORB | Combined | 0.893 | 0.273 | 0.903 | 0.254 | 0.668 (0.639-0.696) |
|  | White | 0.883 | 0.281 | 0.901 | 0.247 | 0.664 (0.633-0.691) |
|  | Black | 0.930 | 0.239 | 0.910 | 0.293 | 0.679 (0.605-0.751) |
|  |  |  |  |  |  |  |
| ATS/IDSA 2007 | Combined | 0.972 | 0.197 | 0.467 | 0.906 | 0.789 (0.765-0.810) |
|  | White | 0.965 | 0.208 | 0.463 | 0.895 | 0.776 (0.750-0.803) |
|  | Black | 0.993 | 0.156 | 0.484 | 0.966 | 0.857 (0.814-0.895) |
|  |  |  |  |  |  |  |
| SMART COP | Combined | 0.909 | 0.215 | 0.746 | 0.481 | 0.670 (0.642-0.697) |
|  | White | 0.898 | 0.220 | 0.743 | 0.462 | 0.660 (0.630-0.690) |
|  | Black | 0.950 | 0.193 | 0.756 | 0.593 | 0.715 (0.642-0.781) |
|  |  |  |  |  |  |  |
| SCAP | Combined | 0.911 | 0.257 | 0.811 | 0.452 | 0.695 (0.667-0.723) |
|  | White | 0.902 | 0.269 | 0.813 | 0.439 | 0.687 (0.655-0.717) |
|  | Black | 0.945 | 0.209 | 0.804 | 0.525 | 0.735 (0.666-0.797) |
|  |  |  |  |  |  |  |
| REA ICU | Combined | 0.924 | 0.153 | 0.367 | 0.791 | 0.627 (0.598-0.655) |
|  | White | 0.916 | 0.163 | 0.362 | 0.788 | 0.616 (0.585-0.645) |
|  | Black | 0.954 | 0.114 | 0.386 | 0.810 | 0.682 (0.606-0.753) |

All values are displayed as median with 95% CI unless otherwise noted.

**e-Table 5. Comparison of ATS/IDSA 2007 criteria between White and Black patients hospitalized with CAP**

| Variable | White | Black | p-value | |
| --- | --- | --- | --- | --- |
| Total n | 6,479 | 1,780 |  |  |
| Minor Criteria, n (%) |  |  |  |  |
| Respiratory rate > 30 breaths/min | 1,081 (16.7) | 311 (17.5) | 0.442 |  |
| PaO_2_ / FiO_2_ ≤ 250 | 1645 (25.4) | 395 (22.2) | 0.006 |  |
| Multilobar infiltrates | 2594 (40.0) | 711 (39.9) | 0.965 |  |
| Altered mental status | 1,211 (18.7) | 276 (15.5) | 0.002 |  |
| Blood urea nitrogen ≥ 20 mg/dl | 3,231 (49.9) | 796 (44.7) | <0.001 |  |
| White blood cell count < 4,000 cells/mm3 | 400 (6.2) | 145 (8.1) | 0.004 |  |
| Platelet count < 100,000 cells/mm3 | 383 (5.9) | 94 (5.3) | 0.341 |  |
| Temperature < 36 degrees C | 117 (1.8) | 25 (1.4) | 0.293 |  |
| Systolic blood pressure < 90 mmHg | 754 (11.6) | 156 (8.8) | 0.001 |  |
| Major Criteria, n (%) |  |  |  |  |
| Need for mechanical ventilation | 931 (14.4) | 272 (15.3) | 0.354 |  |
| Septic shock with need for vasopressors | 441 (6.8) | 137 (7.7) | 0.216 |  |

**e-Table 6. ROC Analysis by race and etiology for risk of in-hospital mortality from community-acquired pneumonia.**

PSI: Pneumonia severity index; CURB-65: Confusion, urea > 7 mmol/L, respiratory rate ≥ 30 breaths/min, blood pressure < 90 (systolic) or < 60 mmHg (diastolic), age ≥ 65; PNA: pneumonia; Sens: sensitivity; Spec, specificity; PPV, positive predictive value; NPV, negative predictive value; AUC: area under the curve; PNA, pneumonia.

| Severity Score | Etiology | Race | Sens | Spec | PPV | NPV | AUC |
| --- | --- | --- | --- | --- | --- | --- | --- |
| PSI | Overall | Combined | 0.981 | 0.108 | 0.436 | 0.891 | 0.782 (0.763 – 0.801) |
|  | (n=8,259) | White | 0.981 | 0.111 | 0.415 | 0.903 | 0.786 (0.765 – 0.806) |
|  |  | Black | 0.980 | 0.091 | 0.508 | 0.830 | 0.762 (0.712 – 0.808) |
|  |  |  |  |  |  |  |  |
|  | Bacterial PNA | Combined | 0.985 | 0.097 | 0.378 | 0.920 | 0.820 (0.767 – 0.865) |
|  | (n=1,105) | White | 0.982 | 0.091 | 0.377 | 0.900 | 0.801 (0.743 – 0.855) |
|  |  | Black | 1.000 | 0.127 | 0.380 | 1.000 | 0.904 (0.824 – 0.958) |
|  |  |  |  |  |  |  |  |
|  | Non-SARS-CoV-2  Viral PNA | Combined | 0.987 | 0.056 | 0.472 | 0.833 | 0.848 (0.714 – 0.943) |
|  | (n=334) | White | 0.983 | 0.064 | 0.441 | 0.833 | 0.846 (0.712 – 0.947) |
|  |  | Black | 1.000 | 0.000 | 0.607 | n/a | 0.902 (0.825 – 0.958) |
|  |  |  |  |  |  |  |  |
|  | SARS-CoV-2 PNA | Combined | 0.936 | 0.256 | 0.576 | 0.789 | 0.755 (0.718 – 0.791) |
|  | (n=1,153) | White | 0.935 | 0.270 | 0.558 | 0.807 | 0.759 (0.720 – 0.799) |
|  |  | Black | 0.940 | 0.217 | 0.614 | 0.733 | 0.736 (0.661 – 0.806) |
|  |  |  |  |  |  |  |  |
| CURB-65 | Overall | Combined | 0.965 | 0.137 | 0.676 | 0.674 | 0.735 (0.715 – 0.755) |
|  | (n=8,259) | White | 0.963 | 0.138 | 0.651 | 0.693 | 0.730 (0.709 – 0.752) |
|  |  | Black | 0.969 | 0.128 | 0.768 | 0.582 | 0.741 (0.691 – 0.785) |
|  |  |  |  |  |  |  |  |
|  | Bacterial PNA | Combined | 0.967 | 0.119 | 0.619 | 0.707 | 0.727 (0.670 – 0.780) |
|  | (n=1,105) | White | 0.967 | 0.110 | 0.606 | 0.700 | 0.711 (0.645 – 0.771) |
|  |  | Black | 0.966 | 0.175 | 0.687 | 0.733 | 0.798 (0.699 – 0.878) |
|  |  |  |  |  |  |  |  |
|  | Non-SARS-CoV-2  Viral PNA | Combined | 0.991 | 0.087 | 0.674 | 0.833 | 0.830 (0.695 – 0.931) |
|  | (n=334) | White | 0.988 | 0.099 | 0.651 | 0.833 | 0.819 (0.675 – 0.924) |
|  |  | Black | 1.000 | 0.000 | 0.770 | n/a | 0.799 (0.703 – 0.882) |
|  |  |  |  |  |  |  |  |
|  | SARS-CoV-2 PNA | Combined | 0.909 | 0.321 | 0.779 | 0.571 | 0.745 (0.708 – 0.783) |
|  | (n=1,153) | White | 0.903 | 0.339 | 0.768 | 0.590 | 0.741 (0.698 – 0.786) |
|  |  | Black | 0.921 | 0.268 | 0.803 | 0.512 | 0.751 (0.676 – 0.818) |
|  |  |  |  |  |  |  |  |
| CORB | Overall | Combined | 0.940 | 0.242 | 0.952 | 0.201 | 0.710 (0.688 – 0.730) |
|  | (n=8,259) | White | 0.936 | 0.247 | 0.950 | 0.202 | 0.711 (0.687 – 0.733) |
|  |  | Black | 0.954 | 0.221 | 0.960 | 0.196 | 0.695 (0.641 – 0.749) |
|  |  |  |  |  |  |  |  |
|  | Bacterial PNA | Combined | 0.946 | 0.219 | 0.927 | 0.280 | 0.709 (0.652 – 0.768) |
|  | (n=1,105) | White | 0.948 | 0.203 | 0.927 | 0.267 | 0.685 (0.611 – 0.749) |
|  |  | Black | 0.939 | 0.294 | 0.928 | 0.333 | 0.818 (0.699 – 0.907) |
|  |  |  |  |  |  |  |  |
|  | Non-SARS-CoV-2  Viral PNA | Combined | 0.971 | 0.158 | 0.950 | 0.250 | 0.807 (0.676 – 0.901) |
|  | (n=334) | White | 0.965 | 0.188 | 0.950 | 0.250 | 0.806 (0.675 – 0.905) |
|  |  | Black | 1.000 | 0.000 | 0.951 | n/a | 0.821 (0.701 – 0.913) |
|  |  |  |  |  |  |  |  |
|  | SARS-CoV-2 PNA | Combined | 0.863 | 0.431 | 0.962 | 0.160 | 0.696 (0.654 – 0.736) |
|  | (n=1,153) | White | 0.851 | 0.468 | 0.962 | 0.165 | 0.704 (0.657 – 0.749) |
|  |  | Black | 0.890 | 0.333 | 0.961 | 0.143 | 0.668 (0.579 – 0.754) |
|  |  |  |  |  |  |  |  |
| ATS/IDSA 2007 | Overall | Combined | 0.984 | 0.137 | 0.575 | 0.881 | 0.821 (0.802 – 0.838) |
|  | (n=8,259) | White | 0.982 | 0.140 | 0.568 | 0.870 | 0.809 (0.788 – 0.830) |
|  |  | Black | 0.993 | 0.121 | 0.599 | 0.930 | 0.874 (0.835 – 0.906) |
|  |  |  |  |  |  |  |  |
|  | Bacterial PNA | Combined | 0.994 | 0.122 | 0.498 | 0.960 | 0.818 (0.768 – 0.859) |
|  | (n=1,105) | White | 0.993 | 0.115 | 0.492 | 0.950 | 0.796 (0.738 – 0.843) |
|  |  | Black | 1.000 | 0.161 | 0.530 | 1.000 | 0.901 (0.824 – 0.954) |
|  |  |  |  |  |  |  |  |
|  | Non-SARS-CoV-2  Viral PNA | Combined | 0.995 | 0.080 | 0.606 | 0.917 | 0.884 (0.778 – 0.963) |
|  | (n=334) | White | 0.994 | 0.094 | 0.594 | 0.917 | 0.884 (0.768 – 0.962) |
|  |  | Black | 1.000 | 0.000 | 0.656 | n/a | 0.902 (0.830 – 0.955) |
|  |  |  |  |  |  |  |  |
|  | SARS-CoV-2 PNA | Combined | 0.974 | 0.283 | 0.570 | 0.917 | 0.866 (0.835 – 0.893) |
|  | (n=1,153) | White | 0.967 | 0.298 | 0.568 | 0.904 | 0.855 (0.816 – 0.888) |
|  |  | Black | 0.989 | 0.247 | 0.575 | 0.956 | 0.905 (0.854 – 0.945) |
|  |  |  |  |  |  |  |  |
| SMART COP | Overall | Combined | 0.951 | 0.181 | 0.853 | 0.426 | 0.701 (0.679 – 0.724) |
|  | (n=8,259) | White | 0.948 | 0.183 | 0.848 | 0.422 | 0.698 (0.673 – 0.723) |
|  |  | Black | 0.964 | 0.169 | 0.871 | 0.444 | 0.713 (0.660 – 0.765) |
|  |  |  |  |  |  |  |  |
|  | Bacterial PNA | Combined | 0.966 | 0.163 | 0.765 | 0.627 | 0.761 (0.701 – 0.816) |
|  | (n=1,105) | White | 0.962 | 0.138 | 0.755 | 0.567 | 0.731 (0.660 – 0.795) |
|  |  | Black | 0.986 | 0.302 | 0.819 | 0.867 | 0.883 (0.762 – 0.957) |
|  |  |  |  |  |  |  |  |
|  | Non-SARS-CoV-2  Viral PNA | Combined | 0.973 | 0.100 | 0.888 | 0.333 | 0.702 (0.538 – 0.841) |
|  | (n=334) | White | 0.967 | 0.133 | 0.900 | 0.333 | 0.707 (0.550 – 0.851) |
|  |  | Black | 1.000 | 0.000 | 0.836 | n/a | 0.883 (0.759 – 0.961) |
|  |  |  |  |  |  |  |  |
|  | SARS-CoV-2 PNA | Combined | 0.869 | 0.389 | 0.932 | 0.235 | 0.668 (0.625 – 0.709) |
|  | (n=1,153) | White | 0.855 | 0.385 | 0.928 | 0.222 | 0.669 (0.618 – 0.716) |
|  |  | Black | 0.900 | 0.400 | 0.941 | 0.273 | 0.658 (0.572 – 0.741) |
|  |  |  |  |  |  |  |  |
| SCAP | Overall | Combined | 0.949 | 0.233 | 0.910 | 0.362 | 0.733 (0.712 – 0.756) |
|  | (n=8,259) | White | 0.947 | 0.246 | 0.909 | 0.369 | 0.734 (0.712 – 0.757) |
|  |  | Black | 0.959 | 0.181 | 0.914 | 0.327 | 0.719 (0.664 – 0.775) |
|  |  |  |  |  |  |  |  |
|  | Bacterial PNA | Combined | 0.959 | 0.198 | 0.854 | 0.493 | 0.730 (0.669 – 0.795) |
|  | (n=1,105) | White | 0.958 | 0.178 | 0.851 | 0.467 | 0.710 (0.635 – 0.778) |
|  |  | Black | 0.960 | 0.300 | 0.873 | 0.600 | 0.816 (0.696 – 0.913) |
|  |  |  |  |  |  |  |  |
|  | Non-SARS-CoV-2  Viral PNA | Combined | 0.974 | 0.154 | 0.932 | 0.333 | 0.816 (0.668 – 0.917) |
|  | (n=334) | White | 0.969 | 0.211 | 0.943 | 0.333 | 0.817 (0.665 – 0.920) |
|  |  | Black | 1.000 | 0.000 | 0.885 | n/a | 0.817 (0.698 – 0.913) |
|  |  |  |  |  |  |  |  |
|  | SARS-CoV-2 PNA | Combined | 0.869 | 0.463 | 0.955 | 0.215 | 0.736 (0.695 – 0.779) |
|  | (n=1,153) | White | 0.858 | 0.483 | 0.953 | 0.216 | 0.749 (0.703 – 0.795) |
|  |  | Black | 0.896 | 0.409 | 0.957 | 0.209 | 0.702 (0.610 – 0.789) |
|  |  |  |  |  |  |  |  |
| REA ICU | Overall | Combined | 0.962 | 0.108 | 0.556 | 0.707 | 0.688 (0.666 – 0.709) |
|  | (n=8,259) | White | 0.960 | 0.114 | 0.546 | 0.719 | 0.688 (0.665 – 0.711) |
|  |  | Black | 0.967 | 0.084 | 0.591 | 0.649 | 0.675 (0.617 – 0.729) |
|  |  |  |  |  |  |  |  |
|  | Bacterial PNA | Combined | 0.973 | 0.100 | 0.461 | 0.827 | 0.720 (0.666 – 0.773) |
|  | (n=1,105) | White | 0.967 | 0.089 | 0.444 | 0.783 | 0.701 (0.635 – 0.765) |
|  |  | Black | 1.000 | 0.167 | 0.548 | 1.000 | 0.829 (0.751 – 0.891) |
|  |  |  |  |  |  |  |  |
|  | Non-SARS-CoV-2  Viral PNA | Combined | 0.980 | 0.058 | 0.596 | 0.667 | 0.702 (0.583 – 0.813) |
|  | (n=334) | White | 0.975 | 0.069 | 0.586 | 0.667 | 0.701 (0.576 – 0.811) |
|  |  | Black | 1.000 | 0.000 | 0.639 | n/a | 0.829 (0.758 – 0.895) |
|  |  |  |  |  |  |  |  |
|  | SARS-CoV-2 PNA | Combined | 0.899 | 0.240 | 0.658 | 0.594 | 0.675 (0.632 – 0.719) |
|  | (n=1,153) | White | 0.895 | 0.264 | 0.649 | 0.624 | 0.680 (0.633 – 0.724) |
|  |  | Black | 0.907 | 0.176 | 0.678 | 0.500 | 0.652 (0.560 – 0.740) |
|  |  |  |  |  |  |  |  |

**e-Table 7. Sensitivity Analysis: ROC Analysis by race for risk of direct ICU admission from community-acquired pneumonia, patient subset with arterial blood gases obtained (n=3183)**

PSI: Pneumonia severity index; CURB-65: Confusion, urea > 7 mmol/L, respiratory rate ≥ 30 breaths/min, blood pressure < 90 (systolic) or < 60 mmHg (diastolic), age ≥ 65; PNA: pneumonia; Sens: sensitivity; Spec, specificity; PPV, positive predictive value; NPV, negative predictive value; AUC: area under the curve; PNA, pneumonia.

| Severity Score | Race | Sens | Spec | PPV | NPV | AUC |
| --- | --- | --- | --- | --- | --- | --- |
| PSI | Combined | 0.854 | 0.435 | 0.368 | 0.885 | 0.732 (0.713-0.749) |
|  | White | 0.853 | 0.429 | 0.348 | 0.891 | 0.728 (0.707-0.747) |
|  | Black | 0.855 | 0.460 | 0.445 | 0.863 | 0.746 (0.707-0.784) |
|  |  |  |  |  |  |  |
| CURB-65 | Combined | 0.768 | 0.498 | 0.641 | 0.647 | 0.693 (0.675-0.713) |
|  | White | 0.767 | 0.482 | 0.608 | 0.664 | 0.686 (0.665-0.706) |
|  | Black | 0.770 | 0.579 | 0.767 | 0.584 | 0.729 (0.689-0.767) |
|  |  |  |  |  |  |  |
| CORB | Combined | 0.694 | 0.721 | 0.949 | 0.238 | 0.734 (0.717-0.751) |
|  | White | 0.692 | 0.702 | 0.945 | 0.236 | 0.727 (0.708-0.748) |
|  | Black | 0.701 | 0.803 | 0.967 | 0.246 | 0.757 (0.717-0.794) |
|  |  |  |  |  |  |  |
| ATS/IDSA 2007 | Combined | 0.909 | 0.545 | 0.592 | 0.892 | 0.848 (0.833-0.861) |
|  | White | 0.905 | 0.538 | 0.580 | 0.889 | 0.844 (0.829-0.861) |
|  | Black | 0.925 | 0.577 | 0.635 | 0.906 | 0.858 (0.828-0.885) |
|  |  |  |  |  |  |  |
| SMART COP | Combined | 0.763 | 0.653 | 0.848 | 0.522 | 0.772 (0.756-0.788) |
|  | White | 0.763 | 0.650 | 0.845 | 0.522 | 0.767 (0.749-0.786) |
|  | Black | 0.765 | 0.669 | 0.859 | 0.519 | 0.794 (0.757-0.828) |
|  |  |  |  |  |  |  |
| SCAP | Combined | 0.752 | 0.729 | 0.907 | 0.457 | 0.775 (0.758-0.793) |
|  | White | 0.753 | 0.733 | 0.908 | 0.458 | 0.775 (0.755-0.794) |
|  | Black | 0.750 | 0.716 | 0.901 | 0.455 | 0.778 (0.739-0.815) |
|  |  |  |  |  |  |  |
| REA ICU | Combined | 0.803 | 0.438 | 0.432 | 0.807 | 0.687 (0.668-0.707) |
|  | White | 0.804 | 0.437 | 0.426 | 0.812 | 0.691 (0.679-0.713) |
|  | Black | 0.798 | 0.442 | 0.455 | 0.789 | 0.674 (0.630-0.717) |

All values are displayed as median with 95% CI unless otherwise noted.

**e-Table 8. ROC Analysis by race and etiology for risk of direct ICU admission from community-acquired pneumonia**

PSI: Pneumonia severity index; CURB-65: Confusion, urea > 7 mmol/L, respiratory rate ≥ 30 breaths/min, blood pressure < 90 (systolic) or < 60 mmHg (diastolic), age ≥ 65; PNA: pneumonia; Sens: sensitivity; Spec, specificity; PPV, positive predictive value; NPV, negative predictive value; AUC: area under the curve; PNA, pneumonia.

| Severity Score | Etiology | Race | Sens | Spec | PPV | NPV | AUC |
| --- | --- | --- | --- | --- | --- | --- | --- |
| PSI | Overall | Combined | 0.946 | 0.245 | 0.468 | 0.867 | 0.774 (0.760 - 0.787) |
|  | (n=8,259) | White | 0.944 | 0.243 | 0.445 | 0.872 | 0.770 (0.755 - 0.7850 |
|  |  | Black | 0.952 | 0.252 | 0.549 | 0.845 | 0.789 (0.759 – 0.816) |
|  |  |  |  |  |  |  |  |
|  | Bacterial PNA | Combined | 0.944 | 0.372 | 0.455 | 0.923 | 0.809 (0.780 – 0.836) |
|  | (n=1,105) | White | 0.943 | 0.373 | 0.458 | 0.921 | 0.805 (0.775 – 0.834) |
|  |  | Black | 0.952 | 0.364 | 0.444 | 0.935 | 0.827 (0.752 – 0.890) |
|  |  |  |  |  |  |  |  |
|  | Non-SARS-CoV-2 Viral PNA | Combined | 0.974 | 0.206 | 0.512 | 0.902 | 0.855 (0.788 – 0.910) |
|  | (n=334) | White | 0.974 | 0.199 | 0.477 | 0.912 | 0.862 (0.791 – 0.919) |
|  |  | Black | 0.973 | 0.250 | 0.667 | 0.857 | 0.848 (0.709 – 0.955) |
|  |  |  |  |  |  |  |  |
|  | SARS-CoV-2 PNA | Combined | 0.920 | 0.256 | 0.571 | 0.747 | 0.742 (0.702 – 0.779) |
|  | (n=1,153) | White | 0.904 | 0.263 | 0.547 | 0.736 | 0.727 (0.682 – 0.771) |
|  |  | Black | 0.950 | 0.237 | 0.622 | 0.783 | 0.769 (0.688 – 0.842) |
|  |  |  |  |  |  |  |  |
| CURB-65 | Overall | Combined | 0.906 | 0.301 | 0.708 | 0.631 | 0.725 (0.711 – 0.739) |
|  | (n=8,259) | White | 0.904 | 0.293 | 0.681 | 0.648 | 0.717 (0.700 – 0.733) |
|  |  | Black | 0.911 | 0.341 | 0.805 | 0.563 | 0.754 (0.726 – 0.784) |
|  |  |  |  |  |  |  |  |
|  | Bacterial PNA | Combined | 0.865 | 0.443 | 0.697 | 0.689 | 0.744 (0.713 – 0.775) |
|  | (n=1,105) | White | 0.865 | 0.437 | 0.686 | 0.696 | 0.739 (0.705 – 0.775) |
|  |  | Black | 0.864 | 0.476 | 0.756 | 0.652 | 0.769 (0.690 – 0.841) |
|  |  |  |  |  |  |  |  |
|  | Non-SARS-CoV-2 Viral PNA | Combined | 0.954 | 0.270 | 0.713 | 0.756 | 0.797 (0.719 – 0.864) |
|  | (n=334) | White | 0.965 | 0.277 | 0.695 | 0.824 | 0.807 (0.714 – 0.879) |
|  |  | Black | 0.915 | 0.214 | 0.796 | 0.429 | 0.799 (0.672 – 0.894) |
|  |  |  |  |  |  |  |  |
|  | SARS-CoV-2 PNA | Combined | 0.901 | 0.337 | 0.782 | 0.564 | 0.736 (0.696 – 0.773) |
|  | (n=1,153) | White | 0.892 | 0.352 | 0.769 | 0.573 | 0.735 (0.689 – 0.778) |
|  |  | Black | 0.921 | 0.293 | 0.809 | 0.533 | 0.732 (0.650 – 0.807) |
|  |  |  |  |  |  |  |  |
| CORB | Overall | Combined | 0.863 | 0.625 | 0.974 | 0.221 | 0.770 (0.757 – 0.785) |
|  | (n=8,259) | White | 0.859 | 0.607 | 0.971 | 0.219 | 0.767 (0.752 – 0.782) |
|  |  | Black | 0.877 | 0.709 | 0.983 | 0.227 | 0.781 (0.751 – 0.812) |
|  |  |  |  |  |  |  |  |
|  | Bacterial PNA | Combined | 0.791 | 0.781 | 0.974 | 0.262 | 0.797 (0.768 – 0.826) |
|  | (n=1,105) | White | 0.789 | 0.785 | 0.975 | 0.258 | 0.789 (0.755 – 0.821) |
|  |  | Black | 0.799 | 0.765 | 0.970 | 0.283 | 0.842 (0.773 – 0.903) |
|  |  |  |  |  |  |  |  |
|  | Non-SARS-CoV-2 Viral PNA | Combined | 0.911 | 0.684 | 0.980 | 0.317 | 0.777 (0.687 – 0.854) |
|  | (n=334) | White | 0.911 | 0.688 | 0.979 | 0.324 | 0.799 (0.703 – 0.881) |
|  |  | Black | 0.914 | 0.667 | 0.981 | 0.286 | 0.681 (0.466 – 0.868) |
|  |  |  |  |  |  |  |  |
|  | SARS-CoV-2 PNA | Combined | 0.868 | 0.692 | 0.979 | 0.241 | 0.782 (0.744 – 0.818) |
|  | (n=1,153) | White | 0.854 | 0.723 | 0.980 | 0.238 | 0.793 (0.751 – 0.834) |
|  |  | Black | 0.899 | 0.611 | 0.977 | 0.250 | 0.749 (0.660 – 0.824) |
|  |  |  |  |  |  |  |  |
| ATS/IDSA 2007 | Overall | Combined | 0.960 | 0.316 | 0.625 | 0.870 | 0.848 (0.836 – 0.861) |
|  | (n=8,259) | White | 0.957 | 0.316 | 0.617 | 0.866 | 0.846 (0.832 – 0.860) |
|  |  | Black | 0.969 | 0.313 | 0.651 | 0.886 | 0.858 (0.833 – 0.883) |
|  |  |  |  |  |  |  |  |
|  | Bacterial PNA | Combined | 0.955 | 0.447 | 0.602 | 0.920 | 0.876 (0.852 – 0.899) |
|  | (n=1,105) | White | 0.951 | 0.442 | 0.595 | 0.912 | 0.868 (0.841 – 0.893) |
|  |  | Black | 0.977 | 0.473 | 0.637 | 0.957 | 0.916 (0.857 – 0.960) |
|  |  |  |  |  |  |  |  |
|  | Non-SARS-CoV-2 Viral PNA | Combined | 0.964 | 0.246 | 0.645 | 0.829 | 0.854 (0.789 – 0.908) |
|  | (n=334) | White | 0.968 | 0.248 | 0.632 | 0.853 | 0.870 (0.799 – 0.929) |
|  |  | Black | 0.950 | 0.238 | 0.704 | 0.714 | 0.767 (0.624 – 0.888) |
|  |  |  |  |  |  |  |  |
|  | SARS-CoV-2 PNA | Combined | 0.951 | 0.278 | 0.563 | 0.853 | 0.820 (0.783 – 0.853) |
|  | (n=1,153) | White | 0.939 | 0.293 | 0.559 | 0.833 | 0.811 (0.767 – 0.851) |
|  |  | Black | 0.978 | 0.241 | 0.570 | 0.913 | 0.850 (0.787 – 0.905) |
|  |  |  |  |  |  |  |  |
| SMART COP | Overall | Combined | 0.901 | 0.503 | 0.900 | 0.505 | 0.781 (0.767 – 0.795) |
|  | (n=8,259) | White | 0.900 | 0.504 | 0.897 | 0.511 | 0.780 (0.763 – 0.795) |
|  |  | Black | 0.908 | 0.498 | 0.913 | 0.481 | 0.787 (0.755 – 0.818) |
|  |  |  |  |  |  |  |  |
|  | Bacterial PNA | Combined | 0.870 | 0.623 | 0.867 | 0.629 | 0.811 (0.781 – 0.840) |
|  | (n=1,105) | White | 0.869 | 0.614 | 0.861 | 0.629 | 0.808 (0.776 – 0.839) |
|  |  | Black | 0.877 | 0.674 | 0.896 | 0.630 | 0.828 (0.750 – 0.895) |
|  |  |  |  |  |  |  |  |
|  | Non-SARS-CoV-2 Viral PNA | Combined | 0.925 | 0.475 | 0.928 | 0.463 | 0.770 (0.684 – 0.847) |
|  | (n=334) | White | 0.922 | 0.500 | 0.937 | 0.441 | 0.773 (0.673 – 0.859) |
|  |  | Black | 0.941 | 0.400 | 0.889 | 0.571 | 0.783 (0.548 – 0.946) |
|  |  |  |  |  |  |  |  |
|  | SARS-CoV-2 PNA | Combined | 0.879 | 0.583 | 0.953 | 0.333 | 0.773 (0.735 – 0.811) |
|  | (n=1,153) | White | 0.867 | 0.615 | 0.954 | 0.333 | 0.784 (0.742 – 0.826) |
|  |  | Black | 0.907 | 0.500 | 0.951 | 0.333 | 0.739 (0.661 – 0.813) |
|  |  |  |  |  |  |  |  |
| SCAP | Overall | Combined | 0.891 | 0.633 | 0.952 | 0.418 | 0.807 (0.793 – 0.821) |
|  | (n=8,259) | White | 0.889 | 0.635 | 0.951 | 0.420 | 0.804 (0.788 – 0.819) |
|  |  | Black | 0.900 | 0.621 | 0.955 | 0.407 | 0.816 (0.785 – 0.846) |
|  |  |  |  |  |  |  |  |
|  | Bacterial PNA | Combined | 0.840 | 0.743 | 0.941 | 0.486 | 0.842 (0.815 – 0.868) |
|  | (n=1,105) | White | 0.837 | 0.732 | 0.939 | 0.479 | 0.840 (0.809 – 0.868) |
|  |  | Black | 0.854 | 0.800 | 0.956 | 0.522 | 0.856 (0.789 – 0.914) |
|  |  |  |  |  |  |  |  |
|  | Non-SARS-CoV-2 Viral PNA | Combined | 0.916 | 0.577 | 0.962 | 0.366 | 0.820 (0.721 – 0.901) |
|  | (n=334) | White | 0.917 | 0.684 | 0.975 | 0.382 | 0.826 (0.719 – 0.917) |
|  |  | Black | 0.907 | 0.286 | 0.907 | 0.286 | 0.817 (0.660 – 0.942) |
|  |  |  |  |  |  |  |  |
|  | SARS-CoV-2 PNA | Combined | 0.877 | 0.695 | 0.974 | 0.303 | 0.764 (0.723 – 0.804) |
|  | (n=1,153) | White | 0.862 | 0.683 | 0.971 | 0.287 | 0.761 (0.714 – 0.807) |
|  |  | Black | 0.911 | 0.727 | 0.980 | 0.356 | 0.776 (0.688 – 0.856) |
|  |  |  |  |  |  |  |  |
| REA ICU | Overall | Combined | 0.926 | 0.273 | 0.597 | 0.761 | 0.755 (0.739 – 0.768) |
|  | (n=8,259) | White | 0.924 | 0.274 | 0.586 | 0.765 | 0.751 (0.735 – 0.768) |
|  |  | Black | 0.934 | 0.268 | 0.636 | 0.747 | 0.766 (0.733 – 0.795) |
|  |  |  |  |  |  |  |  |
|  | Bacterial PNA | Combined | 0.893 | 0.379 | 0.532 | 0.818 | 0.764 (0.733 – 0.793) |
|  | (n=1,105) | White | 0.902 | 0.381 | 0.523 | 0.838 | 0.772 (0.741 – 0.806) |
|  |  | Black | 0.857 | 0.367 | 0.578 | 0.717 | 0.724 (0.643 – 0.803) |
|  |  |  |  |  |  |  |  |
|  | Non-SARS-CoV-2 Viral PNA | Combined | 0.944 | 0.217 | 0.631 | 0.732 | 0.766 (0.689 – 0.841) |
|  | (n=334) | White | 0.949 | 0.224 | 0.623 | 0.765 | 0.789 (0.708 – 0.858) |
|  |  | Black | 0.923 | 0.182 | 0.667 | 0.571 | 0.676 (0.409 – 0.903) |
|  |  |  |  |  |  |  |  |
|  | SARS-CoV-2 PNA | Combined | 0.899 | 0.268 | 0.667 | 0.620 | 0.700 (0.656 – 0.741) |
|  | (n=1,153) | White | 0.887 | 0.283 | 0.653 | 0.622 | 0.692 (0.644 – 0.736) |
|  |  | Black | 0.925 | 0.227 | 0.695 | 0.614 | 0.721 (0.630 – 0.802) |
|  |  |  |  |  |  |  |  |
